# Letter to the Editor: “Multichannel deep learning prediction of major pathological response after neoadjuvant immunochemotherapy in lung cancer: a multicenter diagnostic study”

**DOI:** 10.1097/JS9.0000000000003109

**Published:** 2025-07-23

**Authors:** Qianru Yang, Li Hou, Jing Wang

**Affiliations:** Dongzhimen Hospital, Beijing University of Chinese Medicine, Beijing, China

*Dear Editor,*

We read with interest the recent article by Geng *et al.* published in the International Journal of Surgery, which introduces a multichannel deep learning model for predicting major pathological response (MPR) following neoadjuvant immunochemotherapy in non-small cell lung cancer[1]. Accurate preoperative MPR prediction is essential for optimizing surgical strategies and individualizing patient care. The authors’ innovative Transformer-enhanced approach significantly advances this important clinical need. Nevertheless, we believe that several important aspects warrant further discussion. The letter to the editor is compliant with the TITAN Guidelines 2025—governing declaration and use of AI[2].

Firstly, while authors acknowledge inherent limitations such as retrospective design, limited sample size, and restricted clinical information, one critical issue is baseline cohort differences. As shown in Table 1, statistically significant differences exist between training and test cohorts in smoking (*P* = 0.017), N stage (*P*= 0.041), location of lesion (*P*= 0.039), and pathological type (*P* = 0.019)[1]. These discrepancies introduce a domain shift that could compromise the model’s robustness and limit its applicability across diverse patient populations and imaging protocols[3]. Future studies should prioritize training on larger, prospective multi-institutional datasets and consider incorporating domain adaptation strategies to improve generalizability.

Secondly, while MPR is an important and clinically relevant outcome, the choice of this endpoint complicates direct comparisons with other state-of-the-art models that predominantly evaluate pathological complete response[4]. The distinction in outcome definitions may hinder benchmarking efforts and cross-study interpretability. In addition, the model focused solely on pre-treatment CT imaging, whereas the NeoPred study emphasized the potential value of capturing the temporal changes in tumor features[5]. Future work could explore the integration of multi-time-point image data into the Transformer fusion framework.

In summary, we commend the authors for their innovative and timely work. Addressing the outlined concerns could further enhance the model’s clinical applicability and translational impact.

## Data Availability

All data are included in the article.
